
JOURNAL INFORMATION
==============================
NLM Title Abbreviation: Br J Cancer
Journal ID: Br J Cancer
NLM Journal ID: 0370635

British Journal of Cancer

ISSN: 0007-0920
EISSN: 1532-1827
Publisher: Nature Publishing Group

ARTICLE INFORMATION
==============================
PMCID: PMC2376284
DOI: 10.1038/sj.bjc.6600720
Article ID: 6600720
Article version: 1
Subjects: Author Index for Volume 87

Author Index

Print publication date: 2002 Dec 2
Electronic publication date: 2002 Dec 2
Volume: 87
Issue: 12
First page: 1482
Last page: 1487
Copyright: Copyright © 2002 Cancer Research UK
Copyright year: 2002
Copyright holder: Cancer Research UK
License: This article is licensed under a Creative Commons Attribution 4.0 International License, which permits use, sharing, adaptation, distribution and reproduction in any medium or format, as long as you give appropriate credit to the original author(s) and the source, provide a link to the Creative Commons license, and indicate if changes were made.The images or other third party material in this article are included in the article’s Creative Commons license, unless indicated otherwise in a credit line to the material.If material is not included in the article’s Creative Commons license and your intended use is not permitted by statutory regulation or exceeds the permitted use, you will need to obtain permission directly from the copyright holder. To view a copy of this license, visit https://creativecommons.org/licenses/by/4.0/.
License URL: https://creativecommons.org/licenses/by/4.0/

==============================

British Journal of Cancer (2002) 87, 1482 – 1487
© 2002 Cancer Research UK All rights reserved 0007 – 0920/02 $25.00
www.bjcancer.com

### Author Index for Volume 87

Abd Ellatif, M 1294
Abe T, 1153
Abeler, V 175
Aboagye, EO 783
Ackland, S 846
Adamson, KL 600
Addo S, 1354
Aft RL, 805
Agrez, MV 348
Aguirre, A 414
Agúndez, JAG 681
Ah-Weng, A 1386
Ahmad, SA 1182
Airoldi, L 1230
Akhmedkhanov, A 49
Akre, O 545
Al-Sakkaf, KA 909
Albanes, D 960
Alexander, FE 513
Allen, SG 1257
Alston, RD 1267
Altieri, A 1227
Amadori, D 868
Ameri, K 1173
Andachi, H 441
Andersen, MT 994
Andersen, SN 756
Anderson, A 1365
Anderson, TJ 334
Andreoni, M 1253
Andreyev, HJN 134
Arance, A 562
Arbea, L 158
Ardern-Jones, 905
Arends, MJ 1162
Arkenau, HT 702
Armstrong, S 268
Arndt, O 938
Arndt, V 519
Arslan, A 324, 1417
Ashcroft, L 562
Aso, T 377
Assadourian, S 1210
Ateenyi-Agaba, C 301
Atkin, SL 86
Atkinson, AD 381
Aumann, J 1328
Auvinen, A 511
Avizienyte, E 1128
Avril, A 615
Azli, N 1072

Babb, P 1267
Bachelot, T 1079
Baeten, CIM 344
Baeyens, A 1379
Bafaloukos, D 181
Baguley, BC 465
Bahlitzanakis, N 385
Bahlmann, F 635
Bailey, S 1092
Baillet, M 161
Baker, P 8
Balakrishnan, A 98
Balasubramanian, SP 1057
Ballini, J-P 1470
Ballmer-Hofer, K 106
Baracos, VE 1370
Barker, KT 446
Barnes, DM 208
Barnett, AA 246
Barraclough, R 423
Barratt, A 502
Barshishat, M 1314
Bartlett, JMS 654
Basso, O 524
Bateman, AC 834
Batley, SJ 1162
Batlle, A 471, 790
Baxter, K 733
Beare, S 15
Becker, JC 840
Becker, M 1328
Beckers, TL 924
Beex, LVAM 772
Begent, RHJ 600
Behr, Th 711
Beijnen, JH 608
Bekkers, RLM 373
Bell, BA 621
Bellocco, R 545
Benharroch, D 1314
Bennedsgaard, KM 194
Benner, A 1431
Benoy, I 1437
Beral, V 301
Beravs, K 1047
Berger, C 1197
Berghmans, T 694
Bermúdez Moretti, M 471
Bernard, JL 1197
Berney, DM 624
Bernstein, L 28
Bertuzzi, M 516, 956
Bevan, S 446, 1445
Bezemer, PD 75
Bhagavathula, N 457
Bianchi, S 65
Bidgood, C 733
Biglia, N 1287, 1294
Bijker, N 615
Birch JM 513, 1267
Biron, P 1079
Bishop, K 1445
Bisset, D 1072
Bissonnette, RP 555
Bjørge, L 1119
Bjørge, T 61
Björkstén, B 989
Blackhall, FH 381
Blackwell, RP 1257
Blagden, S 716
Blakey, D 600
Blant, SA 1470
Blay, JY 1079
Blesing, C 716
Boeing, H 519
Bokemeyer, C 729, 1066
Bonithon-Kopp, C 400
Bonneterre, J 1210
Boonstra, H 373
Borgoño, CA 1287
Bosetti, C 516, 956, 1227, 1230
Boshoff, C 301
Bouchier-Hayes, DJ 231
Boulamatsis, D 21
Boulay, J-L 630
Bourboulia, D 301
Bousarghin, L 301
Boutell, J 1162
Boutou, O 740
Bouvier, AM 400
Boven, E 665
Boyd, NF 876
Boyle, P 291
Brachetti, C 635
Bramhall, SR 161
Brant, R 1086
Bray, I 43
Breda, M 608
Brennan, P 43
Brenner, B 1404
Brenner, H 519
Bresnahan, B 1341
Bridge, JA 446
Brimmell, M 834
Brison, DR 381
Brock, C 1092
Bröcker, E-B 840
Brown, BL 909
Brown, NJ 1057
Brown, DJF 264
Brown, PD 161
Brown, SB 246
Brown, VL 208
Browne, J 476
Bruera, E 1370
Brunton, VG 1128
Brynes, RK 28
Bu, X 28
Bucana, CD 1182
Buccheri, G 1112
Buckels, JAC 161
Bullock, S 1445
Bundred, N 691
Burgers, JA 562
Burke, B 1173
Butow, PN 502
Bützow, R 1119
Byrne, MJ 491

Caca, K 689
Caine, GJ 1337
Caldas, C 1162
Califano, D 1479
Calvo, E 158
Cambeiro, M 158
Camero, A 129
Cameron, DA 1365
Campbell, NC 585
Cardar, PJ 687
Carpenter, L 301
Carrasco, R 432
Carroll, M 537
Cartwright, JE 673
Casabonne, D 301
Casas, A 471, 790
Cassidy, J 585
Cataliotti, L 615
Cella, D 1341
Cemazar, M 1047
Cerutti, J 1479
Chang, T-T 1449
Chang, Y-C 1449
Chapusot, C 400
Charlotte, F 1390
Charnley, RM 1034
Cheeseman, SL 393
Chen, C-J 966
Chen, HH-W 1449
Chen, R 1166
Chen, Y-F 1000
Chen, S-H 359
Chen, Y-L 359
Cherian, MG 1019
Chester, D 393
Chetty, U 8
Chhajlani, V 414
Chiarotti, L 1479
Ching, L-M 465
Chow, N-H 1449
Chow, W 281
Christiansen, M 994
Chung, H-K 405
Chung, J-H 405
Chung, SCS 91
Cini, G 497
Claes, K 1379
Clandinin, MT 1370
Clarke, PA 567
Clarke, SJ 277
Clausen, N 994
Cnattingius, S 974
Coer, A 1047
Colgan, T 763
Collaborative Group on Hormonal Factors in Breast Cancer, 1234
Collan, Y 1275
Colowick, AB 268
Colston, KW 555, 1340
Connolly, EM 231
Coppes, MJ 1086
Coradini, D 1105
Corfe, F 733
Correa García, S 471
Correale, P 720
Corrie, P 716
Cortes, J 158
Cosaert, J 825
Cotter, TG 1188
Coursaget, P 301
Coyle, P 533
Coze, C 1197
Craanen, ME 892
Craft, AW 1092

Cresswell, J 1034
Crocetti, E 65
Crown, J 1365
Cruz, T 1253
Cugnenc, P-H 551
Cull, A 1099
Curtin, JF 1188
Cutress, RI 834

Dai, Y-C 1449
Daidone, MG 1105
Daigo, Y 1162
Dal Maso, L 1227
Dalgleish, AG 555, 1166
Damato, BE 1308
D'Ath, S 716
Davies, DC 621
Day, NE 1257
De Bruijn, P 144
de Graaf, M 659
de Jong, D 892
De Luca Cardillo, C 497
De Manzoni, G 171
de Mascarel, I 70
de Nigris, F 1479
De Paola, F 868
De Ridder, L 1379
Dearnaley, DP 905
DeCatris, M 15
Deichmann, M 1431
Delmotte, P 694
Delvaux, N 1
Dent, JT 393
Denton, AS 134
Di Leo, A 171
Diamandis, EP 339, 763, 1287, 1294
Diamandis, P 763
Diamond, EJ 726
Diaz-Gonzalez, JA 158
Dickinson, HO 746
Diep, A 294
Diep, CB 756
Dietz, A 938
Dillner, J 61, 175
Dirix, LY 1437
Distefano, MG 1145
Dixon, JM 8, 334, 950
Dobson, PRM 909
Domizio, P 779
Dommering, CJ 892
Done, K 733
Doni, L 497
Donskov, F 194
Dorahy, DJ 348
Doroshow, JH 281
dos Santos Silva, I 1195
Dosaka-Akita, H 751
Dredge, K 1166
Droz, JP 1079
Dubini, A 868
Dubus, L 1
Duffy, SW 65
Dumortier, A 1079
Dünne, A-A 711
Dunn, I 654
Durieux, J-F 1
Dyckhoff, G 938

Easton, DF 905
Eatough, JP 509
Ebert, MPA 91
Ecarnot-Laubriet, A 400
Edwards, SM 905
Eeles, RA 905, 1445
Efferth, T 251
Egeler, RM 1086
Egner, U 1431
Egrie, J 476
Ehlin, AGC 989
Ekbom, A 974, 989
Ekbom, A 545
Elliott, AM 585
Ellis, LM 1182
Elomaa, I 591
Endo, T 918
Engeland, A 61
Epoetin Alfa Study Group, 1341
Erlandsson, G 974
Ernst, E 479
Escobar, A 414
Esumi, H 898
Evans, TRJ 1365
Evans, S 567

Faivre, J 400
Falk, RT 54
Fallowfield, L 854, 1341
Fan, F 1182
Fan, LZ 1019
Fargeot, P 1210
Farvacques, C 1
Fears, TR 54
Fedier, A 1027
Fenig, E 1404
Fentiman, IS 615
Fernandez, L 1253
Ferrandina, G 1145
Ferrigno, D 1112
Fiaschi, AI 720
Fichtner, I 1328
Field, CJ 1370
Field, JK 1308
Fielding, D 491
Fielding, R 982
Fink, D 1027
Finlayson, CJ 446
Fioretto, LM 497
Fishell, E 876
Fisher, C 446
Fisker, R 194
Flanagan, AM 446
Fodstad, Ø 1281
Foekens, JA 772
Foley, D 231
Folz, BJ 711
Foo, W 982
Forbes, A 134
Forbes, K 733
Forman, S 281
Forrest, LM 264
Forsea, A-M 366
Forster, T 8
Försti, A 1162
Forsyth, A 654
Fouret, P 1390

Fracchioli, S 763
Fraier, D 608
Frame, MC 1128
Franceschi, S 314, 324, 516, 1227, 1230, 1253, 1417
Francini, G 720
Francis, RJ 600
Frangia, K 181
Frankel, P 281
Frappaz, D 1197
Frassineti, GL 868
French, JJ 1034
Frerichs, J 113
Freund, M 1341
Friery, OP 1339
Fritz, G 635
Froudarakis, M 385
Frye, RA 1479
Fujimori, T 1042
Fujino, Y 37
Fujisawa, T 81
Fujita, Y 1153
Fukuda, H 258, 790
Fukunaga, A 1140
Fukuoka, M 258
Fulignati, C 497
Funada, Y 1136
Furuhata, S 1454
Fusco, A 1479
Fusenig, NE 1301

Gaff, C 502
Gaffney, J 624
Gagnon, D 1341
Gallagher, CJ 850
Gallagher, R 1339
Gallus, S 516, 956, 1227
Gambi, A 868
Game, PA 533
Gao, DL 977
Garcia-Foncillas, J 158
García-Gamito, FJ 681
García-Martín, E 681
Garzon, C 158
Gazdar, AF 432
Geilen, CC 366
George, WD 1246
Gerritsen, WR 659
Gessi, M 1145
Geurti-Lackner, B 1445
Geurts-Moespot, J 772
Ghali, L 319
Giardina, G 763
Gil-Bazo, I 158
Gille, JJP 892
Giorgi, D 65
Giridharan, P 98
Gishizki, M 645
Gislefoss, RE 61
Gisselsson, D 202
Gius, D 805
Glaholm, J 938
Glanzmann, TM 1470
Glaspy, JA 268
Gnekow, A 945
Go, MYY 91
Goff, LK 779
Goff, L 537

Gogas, H 181
Gomez, F 1079
Gorzegno, G 1341
Goto, K 1360
Granato, AM 868
Grazia Porro, M 608
Greco, A 645
Green, AJ 600
Gregor, M 702
Greschniok, A 702
Grieve, RJ 1365
Griffioen, AW 344
Griffiths, K 1246
Griffiths, J 246
Grigor, KM 654
Grossbard, ML 372
Groves, F 511
Gu, X 348
Guastalla, JP 1079, 1210
Guizard, A-V 740
Gumbrell, L 600
Gurpide, A 158
Gyselman, VG 537

Hagiwara, A 1153
Hague, A 834
Haisma, HJ 659
Hakulinen, J 1119
Hall, CN 168
Hall, GH 86
Hall, K 846
Haller, U 1027
Hamburger, AW 1396
Hamilton, AS 294
Hamilton-Dutoit, S 1422
Hammel, P 551
Hammond, G 876
Hanks, GW 733
Hanselaar, AGJM 373
Harada, M 796, 1006
Harmey, JH 231
Harris, AL 1173
Harris, J 567
Harrison, KL 168
Hart, AAM 615
Hart, IR 859
Hartmann, JT 1066
Hartschuh, W 1301, 1431
Harwood, C 319
Hasan, T 1321
Hashimoto, D 1454
Hasle, H 524
Hass, HG 702
Hata, H 81
Hawkins, RA 8
Hayakawa, N 309
Hayase, H 1454
Hayashizaki, Y 1153
Hebert, C 1396
Hedley, AJ 982
Heighway, J 1308
Helboe, L 86
Helmerhorst, TJM 75
Henshaw, DL 1336
Hentrich, M 729
Herrera, I 339
Herrmann, R 630
Hicks, DJ 352

© 2002 Cancer Research UK

Hida, N 796
Hill, E 168
Hillen, HFP 344
Hilson, AJ 600
Hino, S 1042
Hiraoka, K 1140
Hirst, DG 1339
Hitomi, Y 898
Hjalgrim, H 994
Hjalgrim, LL 994
Ho, C-K 529
Ho, C-L 1449
Ho, S-M 1411
Hochhauser, D 600, 1216
Hodgetts, 562
Hoefnagel, CA 705
Hoffman, BR 763
Hofmann, U 151
Hogenraad, R 1
Hogervorst, FBL 892
Hohenberger, P 478
Hoijer, M 113
Hokland, M 194
Hokland, P 994
Hoogsteen, IJ 665
Hoover, RN 54
Hope-Stone, LD 600
Horiot, J-C 478
Horne, G 381
Horsman, MR 1422
Hoshiyama, Y 309
Hosoda, A 441
Houlston, RS 446, 905, 1445
Hövelmann, S 924
Hoylaerts, M 1437
HPV Study Group, 324, 1417
Hsieh M-C, 1000
Hsu, H-K 529
Hu, J 218
Huget, Ph 1437
Hughes, CM 1339
Hulme, MJ 8
Hummel, M 1462
Hunt L, 1204
Hutchinson, OC 783
Huzarski, T 888
Hwang, J-H 187

Ichikawa, Y 1153
Ikarashi, Y 1454
Ikeguchi, M 883
Imamura, K 898
Ioannovich, J 181
Ishikawa, T 1153
Iskander, L 1287
Ito, H 441
Ito, M 1006
Itoh, K 796, 1006
Itoh, T 751, 1140
Ivanusa, T 1047
Iyer, NG 1162
Izumi, H 751

Jack, WJL 8
Jackson, RA 905
Jackson, S 319
Jacobs, IJ 537
Jadeja, JS 268
Jaffe, H 301
Jager, D 113
Jakubowska, A 888
Jalava, P 1275
James, R 1216
Jamieson, GG 533
Jansen, S 608
Japan Collaborative Cohort Study Group, 309
Jarva, H 1119
Jellum, E 61, 175
Jenkins, V 854
Jensen, JJ 194
Jiang, C-C 218
Jin, C 202
Jin, Y 202
Jobo, T 81
Joel, SP 393
Joensuu, H 591
Jonat, W 1480
Jones, A 1365
Jones, RJ 1128
Jong, R 876
Jonson, T 202
Jordan, S 624
Jordan, VC 449
Jørgensen, P 994
Julien, J-P 615
Jung, EG 151
Junghahn, I 1328
Junnikkala, S 1119
Justice, G 268

Kaibara, N 441, 883
Kaina, B 635
Kaiser, SA 449
Kakinuma, R 1360
Kamata, Y 81
Kameya, T 81
Kämpgen, E 840
Kamura, T 796, 1006
Kanai, T 81
Kanelopoulos, P 21
Kanz, L 729, 1066
Kao, E-L 529
Karthikeyan, NP 98
Katagiri, K 796
Kato, K 898, 1140
Katoh, H 751, 1140
Katsaros, D 763
Kawaguchi, K 441
Kawaguchi, T 309
Kawakami, N 31
Kawamata, H 1042
Kawasaki, H 441
Kay, E 231
Kaye, SB 813, 1072
Kefala, G 181
Kehoe, ST 1337
Kehri, W 938
Kelleher, DK 1462
Kelsell, DP 208
Kelsey, AM 1267
Kennedy, ICS 1365
Kessler, J 268
Kestell, P 465
Kiely, MA 1099
Kiessling, R 414
Kikuchi, R 1136
Kikuchi, S 309
Kilkarni, R 1092
Killen, ME 8
Kim, C-W 405
Kim, D-J 405
Kim, I-J 187
Kim, JI 314
Kim, JY 314
Kim, S-W 187
Kim, W-H 187
Kingston, JE 779
Kinoshita, I 751
Kirby, JA 1034
Kirk, J 502
Kishimoto, Y 441
Kitamura, A 441
Klaproth, H 729
Kleeman, H-W 238
Klemenz, R 106
Kliakhandler, IL 449
Klump, B 702
Knap, MM 1422
Koda, M 441
Koenig, KL 49
Koetsier, M 113
Kogut, N 281
Kohlhagen, G 665
Kohno, K 751
Kollmannsberger, C 729
Kolonel, LN 54
Kondo, S 1140
Kondo, T 309
Kong, X 519
Koomägi, R 251
Korenblit, C 414
Koskela, P 61
Kosmas, C 21
Kostourou, V 673
Kotnik, V 1047
Koukourakis, GV 385
Koukourakis, MI 385
Kouzarides, T 1162
Kreienberg, R 519
Krishna, S 621
Kronqvist, P 1275
Kroon, BBR 705
Kruyt, FA 659
Krzic, M 1047
Ku, J-L 187
Kubin, T 729
Kubota, K 1360
Kubota, T 377
Kuczyk, M 729
Kudoh, S 258
Kuehl, J 945
Kuopio, T 1275
Kuramoto, H 81
Kurihara, H 171
Kyrgias, G 385

La Vecchia, C 291, 516, 956, 1230, 1272
Lacau-St-Guily, J 1390
Laffer, U 630
Lafitte, J-J 694
Lagiou, P 956
Lagrange, M 630
Lai, W-W 1449
Laight, A 1354
Lam, T-H 982
Lambe, M 974
Lansdown, MRJ 687
Larcombe, I 1204
Larsson, O 414
Latif, Z 654
Lau, EMC 982
Lau, B 218
Laurent Puig, P 400, 551
Lauriola, L 1145
Lavery, KM 938
Le Vay, JH 1365
Leach, L 854
Leavitt, RD 938
Lecher, B 1462
Lecoeur, C 537
Ledermann, JA 1216
Lee, DH 314
Lee, DS 314
Lee, J-M 529
Lee, REJ 1092
Lee, W-Y 1449
Lee, Y-C 529
Lees, NP 168
Legge, F 1145
Lehrer, S 726
Lehtinen, M 61, 175
Lence, J 1253
Leonard, P 1216
Leonard, RCF 1365
Leong, L 281
Leung, GM 982
Leung, WK 91
Levenson, AS 449
Levi, F 291, 516, 1227, 1230
Levi, I 1314
Levi, J 846
Lévy, P 551
Lewis, C 846
Lewis, CE 1173
Leygue, E 1411
Lian, S-T 1000
Lie, AK 175
Lieb, W 840
Lieberman, BA 381
Lieverst, J 608
Lillington, DM 779
Lin, S-R 1000
Lip, GYH 1337
Lippert, BM 711
Little, J 585
Littlewood, TJ 1341
Liu, D 423, 783
Liu, D 423
Lobb, EA 502
Lomax, L 562
Loos, WJ 144
López, M 414
Lothe, RA 756
Loukopoulos, D 181
Lowe, DG 537
Lowy, A 630
Løvig, T 756
Lu, Y-J 446
Lubiński, J 888
Lucchini, F 291

© 2002 Cancer Research UK

Lundqvist, A 414
Luo, L-Y 339
Luostarinen, T 61, 175
Lurie, H 1404
Lynch, P 1099

Mælandsmo, GM 1281
Maaya, MB 381
Macdonald, CD 1166
MacGrogen, G 70
Mack, TM 294
Mackey, J 1370
Madsen, HO 994
Maeda, Y 796, 1006
Maggiano, N 1145
Magklara, A 1294
Maher, EJ 134
Maire, F 551
Maitra, A 432
Makino, M 441
Malfertheiner, P 91
Maliepaard, M 665
Malonne, H 475
Man, H-W 1166
Mandahl, N 202
Manders, P 772
Manganelli, A 720
Mansi, J 1365
Mansi, JL 1340
Manuelian, T 1119
Marchal, S 1
Marcussen, N 194, 1422
Margison, GP 168
Margolin, K 281
Marrelli, D 171
Marriott, JB 1166
Marsden, JR 1386
Marshall, JF 859
Marsili, S 720
Martínez, C 681
Martin, B 694
Martin, J 600
Martin, L 400
Martin, LJ 876
Martin-Algarra, S 158
Martins, C 202
Marty, C 106
Marx, G 846
Mascaux, C 694
Maslanyj, MP 1257
Massobrio, M 763
Massuger, LFAG 373
Mathew, G 533
Mathijssen, RHJ 144
Mathoulin-Pelissier, S 70
Matsumoto, T 1360
Matsuo, K 751
Mattern, J 251
Maune, S 938
Mayer, A 716
Mayer, F 729
Maytin, EV 1321
Mbidde, E 301
Mbulaiteye, S 301
McArdle, CS 264, 1246
McCarty, MF 1182
McElgunn, CJ 892
McGuire, S 1445
McIntyre, MA 8
McKeown, SR 1339
McMillan, DC 264
McNally, RJQ 513, 1267
McNamara, M 281
McNulty, P 1341
McWilliams, DF 567
Mee, TJ 1257
Meert, A-P 694
Meijer, CJLM 75, 324, 1417
Meijer, GA 892
Meijer, HP 113
Meiser, B 502
Meitz, JC 905
Melbye, M 994
Melchers, WJG 373
Meling, GI 756
Memari, N 1294
Menko, FH 892
Meri, S 1119
Mertens, F 202
Messiaen, L 1379
Messinese, S 720
Metzner, B 1066
Micard, S 551
Michaud, DS 960
Michon, J 1197
Mild, G 630
Miller, WR 950
Miller, WR 8, 334, 688
Milroy, R 264
Mini, E 497
Minkin, S 876
Minna, JD 432
Miranda, C 645
Mitchell, G 1445
Mitchell, P 491
Miura, Y 309
Miyamoto, M 1140
Mizoue, T 37, 309
Molano, M 324, 1417
Mölenkamp, G 945
Molina, A 281
Moll R, 711
Monnier, A 1210
Monnier, P 1470
Montgomery, SM 989
Moon, SE 457
Mooney, LM 909
Morelli, C 1479
Moretti, R 497
Morgagni, P 171
Morgan, H 733
Morgan Jr, R 281
Morita, T 1153
Moriyama, H 1136
Mork, J 61
Morris, TM 567
Mössner, J 689
Mott, M 1204
M'Rabet, L 113
Muller, GW 1166
Munoz, N 324, 1417
Murawaki, Y 441
Murkin, A 905
Murphy, LC 1411
Murray, MM 1339
Musk, AW 491
Myers, JC 533

Näher, H 1431
Nagata, C 31
Nakagawa, K 258
Nakakubo, Y 1140
Nakanishi, M 1153
Nakashiro, K 1042
Namer, M 1210
Nanni, O 868
Naredi, P 194
Navarro, V 158
Nederlof, PM 892
Negoro, S 258
Negri, E 291, 516, 956, 1227, 1230
Nehls, O 702
Nej, K 888
Nemunaitis, J 161
Neri, B 497
Neri, D 106
Nesland, JM 1281
Neumann, C 840, 1328
Newsham, A 854
Newton, R 301
Ng, EKW 91
Ng, H-K 218
Ngoan, LT 37
Nicholls, K 854
Nicolas, AI 158
Nieweg, OE 705
Niho, S 1360
Nikitakis, NG 1396
Nishimura, M 751
Nishimura, Y 258
Nishiwaki, Y 1360
Nishizuka, I 1153
Nitsche, A 1328
Niu, Y 1411
Niu, J 348
Niya, F 796
Nobbenhuis, MAE 75
Noguchi, T 1136
Nomura, AMY 54
Nonaka, Y 1006
Nooter, K 144
Nowak, AK 491
Nunn, J 1308
Nyari, TA 746

O'Brien, MER 481
O'Byrene, KJ 15
O'Byrne, J 268
Odermatt, B 106
O'Donnell, D 1321
Oechsle, K 1066
Offersen, BV 1422
O'Grady, T 231
Ohmatsu, H 1360
Ohno, E 81
Ohno, Y 309
Okazaki, Y 1153
Oliver, RTD 624
Oliverio, G 868
Olsen, J 524
Olsen, JH 524
O'Malley, D 600
Omotehara, F 1042
Onadim Z, 779
O'Neill VJ 933
Oosterhoff, D 659
Orenberg, EK 938
Orfanos, CE 366
Orpana, A 591
Ortel, B 1321
Ortiz, RM 1253
Oshikiri, T 1140
Osman, S 783
Österlund, P 591
Otsuka, I 377
Overgaard, J 1422
Owens, DW 1128
Özdağ, H 1162

Paci, E 65
Packham, 834
Paesmans, M 694
Pallisgaard, N 994
Pals, G 892
Panagiotou, P 181
Pang, JC-s 218
Pantalone, D 497
Papadakis, ED 1013
Papadopoulos, MC 621
Paraskeva, C 352
Parikh, AA 1182
Park, J-G 187
Park, Y-H 187
Parker, L 746
Parkin, DM 301
Parmar MKB 815
Pasini, F 171
Patterson, LH 1339
Pauson, AW 1246
Pease, KM 449
Pectasides, D 181
Pedersen, KB 1281
Pellizzaro, C 1105
Pellizzoni, C 608
Pepper, MS 537
Pereira, P 225
Perol, D 1197
Perotti, C 790
Perotti, C 471
Perren, TJ 1365
Perumal, K 98
Peters, TJ 733
Peterse, JL 705
Peterse, JL 615
Petersson, M 414
Petrioli R, 720
Pettersson, F 555
Philcox, JC 533
Philip, I 1079
Philip, T 1197
Piard, F 400
Picot, V 70
Pierotti, MA 645
Pietinen, P 960
Pike, MC 54
Pilz, L 1301
Pinedo, HM 659, 665
Pitchforth, E 1221
Pizarro, RM 681
Plagens, A 212
Playle, LC 352

© 2002 Cancer Research UK

Pluzanska, A 938
Polymeris, M-E 1287
Polyzos, A 181
Pommier, Y 665
Ponder, BAJ 1162
Ponnelle, T 400
Ponzone, R 1287, 1294
Poon, W-S 218
Poppe, B 1379
Porschen, R 702
Posso, H 324, 1417
Pottier, D 740
Povey, AC 168
Pozzessere, D 720
Pratt, VC 1370
Prescott, RJ 8
Press, MF 294
Price, E 779
Price, L 1092
Price, P 716, 783
Proby, CM 208
Pukkala, E 61
Puopolo, M 763

Qian, W 815
Qin, Q 977
Qualtrough, D 352
Quattrone, A 497
Quinn, MJ 1267
Quoix, E 825

Radford, JA 381
Rageot, D 400
Raisova, M 366
Rakowsky, E 1404
Ramaswamy, A 711
Ramazeilles, C 1072
Rana, B 574
Ranelletti, FO 1145
Ranzi, V 645
Rappaport, EB 294
Raschko, J 281
Rating, D 945
Ray, RM 977
Ray-Coquard, I 1079
Razavi, D 1
Rebattu, P 1079
Reed, MWR 1057
Reeves, G 301
Reinmuth, N 1182
Repo, H 591
Retalis, G 385
Reuter, J 630
Reynolds, MA 1396
Rezza, G 1253
Rhim, J-H 405
Ribecco, AS 497
Richards, D 268
Richards, FJ 393
Richiardi, L 545
Richmond, I 86
Rick, O 72
Riebeling, C 366
Rigault de la Longrais, IA 763
Ritchie, LD 585
Rittgen, W 151
Riva, A 1072
Rivory, LP 277
Roagna, R 1287
Robbins, A 600
Robbins, M 733
Roberts, A 1092
Roberts, DJH 246
Robinson, BWS 491
Robinson, SP 673
Roccato, E 645
Roché, H 1072, 1210
Roche-Nagle, G 231
Rochlitz, C 630
Rodriguez, J 158
Rofe, AM 533
Rognum, TO 756
Rohloff, J 689
Romanidis, K 385
Romundstad, PR 289, 971
Ronderos, M 324, 1417
Rosing, H 608
Rosselli del Turco, M 65
Rossi, G 268
Roviello, F 171
Rozen, R 225
Rozendaal, L 75
Rudland, PS 423
Ruefenacht, UB 1027
Russell, E 1221
Russell, ST 580
Ruszniewski, P 551
Rutgers, EJTh 615, 705
Ryan, A 537
Ryan, G 491
Ryder, LP 994
Ryder, WDJ 381
Ryu, Y-S 405

Saadoun, S 621
Sabatino, M 720
Sabbaghian, N 225
Sacca, P 790
Sahmoud, T 950
Sahmoud, T 334
Sajedi, M 1086
Sakakura, C 1153
Sakata, K 309
Salazar-Onfray, F 414
Salgado, R 1437
Salutari, V 1145
Salvestrini, F 720
Sandercock, J 815
Sanders, DSA 1386
Santella, RM 966
Santelli, G 1479
Saragoni, L 171
Sarmati, L 1253
Sasaki, T 918
Sato, H 81
Sato, Y 796
Sauk, JJ 1396
Saunders, PTK 688
Sayer, HG 729
Scambia, G 1145
Scarpi, E 868
Scatizzi, M 497
Schöffski, P 1066
Schadendorf, D 151, 840
Scheffer, GL 665
Schellens, JHM 608
Scheper, RJ 665
Schievella, AR 225
Schlüper, HMM 665
Schleicher, J 1066
Schleucher, N 1066
Schmidt, K 479
Schmidt, M 635, 924
Schmiegelow, K 994
Schoemaker, NE 608
Schofield, J 246
Scholes, AGM 1308
Schoppmeyer, K 689
Schott, H 106
Schouten, JP 892
Schulz, J 161
Schwartz, B 1314
Schwartzberg, L 268
Schwarz, VA 1027
Schwendener, RA 106
Scorilas, A 763, 1287, 1294
Scott, HR 264
Scott, R 348
Scott, RJ 888
Sculier, J-P 694
Segal, A 491
Seifart, C 212
Seifart, U 212
Sekhon, J 225
Selby, PJ 1099
Sena, L 600
Senaratne, SG 1340
Sentjurc, M 1047
Serraino, D 1253
Serrano, A 414
Sersa, G 1047
Seymour, K 1034
Seymour, MT 393, 1216
Shahbakhti, H 600
Shamash, J 850
Shamash, J 624
Sharlin, D 1321
Sharma, RA 15
Sharma, SK 600
Sharp, D 733
Sharp, L 585
Shaw, J 716
Shepherd, JH 537
Shi, W 119
Shibata, S 281
Shichijo, S 1006
Shichinohe, T 1140
Shields, F 15
Shimada, H 1153
Shimizu, A 1454
Shimizu, H 31
Shimomura, K 1153
Shin, S-J 1000
Shin, HR 314
Shinohara, T 1140
Shiota, G 441
Shipley, J 446
Shirakawa, K 1454
Shore, RE 49
Siavash, H 1396
Sibson, DR 423
Siemann, DW 119
Sigstad, E 175
Simpson, HW 1246
Simpson, J 1257
Simpson, JF 281
Sinha, AK 1321
Sinha, D 511
Siris, N 1404
Sismondi, P 1287
Sismondi, P 1294
Skillen, AW 1092
Skinner, J 1257
Skinner, R 1092
Skjærven, R 289, 971
Slama, R 740
Slater, S 850
Slaviero, KA 277
Slevin, ML 850
Sloane, JP 1055
Smeets, RL 113
Smith, A 567
Smith, SL 1308
Snell, L 1411
Sodha, N 1445
Soler-Michel, P 1079
Somasundaram, ST 98
Somlo, E 281
Somlo, G 281
Sone, T 659
Song, DH 574
Soosaipillai, A 339
Souhami, RL 478
Soulitzis, N 1013
Spandidos, DA 1013
Sparén, P 989
Sparreboom, A 144
Speight, PM 859
Speirs, V 687
Spinelli, R 608
Spira, A 740
Spott, C 729
Springer, C 600
Stagger, S 726
Steinmann, A 840
Steward, WP 15
Stewart, ME 938
Stirling, D 1166
Stock, RG 726
Stoeltzing, O 1182
Stone, J 876
Stone, NN 726
Storey, A 319
Stratton, MR 905
Strieth, S 1301
Stuart, M 334, 950
Stürmer, T 519
Suh, K-S 187
Suh, Y 405
Sulkes, A 1404
Sun, C-A 966
Sung, JJY 91
Suthers, GK 502
Suzuoki, M 1140
Svoboda, KM 449
Swart, M 608
Swartz, HM 1047
Sweep, CGJ 772
Sykes, J 733
Szedlak, M 151

Takada, K 127

© 2002 Cancer Research UK

Takagi, T 1153
Takahashi, M 796
Takahashi, S 1153
Takatsuka, N 31
Takeda, K 258
Takeno, S 1136
Talamini, R 516, 1227, 1230
Tamakoshi, A 309
Tanaka, M 258
Tang, M 432
Tanis, PJ 705
Tannapfel, A 689
Tannock, I 478
Tannock, IF 238
Tavani, A 956
Taylor, PR 960
Taylor, R 1445
Tchekmedyian, NS 268
Temam, S 1390
ten Bokkel Huinink, WW 608
Terada, M 1454
Terheyden, P 840
Terracciano, L 630
Terret, C 1072
Tesdale, AL 8
Thach, TQ 982
Thatcher, N 562
The Cancer Research UK/BPG UK Familial Prostate Cancer Study Collaborators, 905
Thews, O 1462
Thierens, H 1379
Thomas, AL 15
Thomas, DB 977
Thomas, GJ 859
Thome, M 1431
Thoresen, S 175
Thoresen, SØ 61
Thorstensen, L 756
Tisdale, MJ 580
Tjan-Heijnen, VCG 772
Todo, S 796
Tokui, N 37, 309
Tong, CY-k 218
Toniolo, P 49
Tookman, A 1480
Torri, V 815
Touzé, A 301
Townsend, PA 834
Toy, E 1365
Toyoshima, H 309
Trichopoulos, D 289, 956, 971
Troncoso, P 432
Tsavaris, N 21
Tsiompanou, E 600
Tsoutsos, D 181
Tsuda, N 1006
Tucker, K 502
Tunstall, RG 246
Turci, D 868
Turkes, A 1246
Turnbull, LW 86
Turrini, M 497
Twelves, CJ 933, 1072

Uchida, D 1042
Uchida, Y 1136
Uganda Kaposi's Sarcoma Study Group, 301
Underwood, MA 654
United Kingdom Childhood Cancer Study Investigators, 1257

Vaccarella, S 1253
Vadiaka, M 21
Valdés Olmos, RA 705
van Beusechem, VW 659
Van Bockstaele, D 1437
van den Bergh, H 1470
van den Brule, AJC 75, 324, 1417
van der Meulen, IH 659
Van der Pol, M 1221
van Ham, MAPC 373
van Hattum, AH 665
van Kesteren, C 608
Van Marck, E 1437
van Schooten, RJ 892
Van Tienoven, TH 772
Van Trappen, PO 537
van't Land, B 113
Varani, J 457
Vasey, PA 1072
Vatten, LJ 971
Vatten, LJ 289
Vaupel, P 1462
Velmurugan, R 98
Veneroni, S 1105
Ventura, L 1105
Verdebout, J-M 694
Verhoeven, ICL 344
Verlato, G 171
Vermeulen, P 1437
Vernillet, L 1072
Vernon, DI 246
Verweij, J 144
Vezzosi, V 65
Viglietto, G 1479
Virtamo, J 960
Virtanen, M 960
Vishwakarma, RA 98
Voitot, H 551
Volm, M 251
Volpi, A 868
Von der Maase, H 194
Von Wichart, P 212
Voorhorst, FJ 75
Vral, A 1379

Wabinga, H 301
Wagner, S 840
Wagnières, G 1470
Wagstaff, 344
Wakasugi, H 1454
Wang, J-Y 1000
Wang-Gohrke, S 519
Wang, HB 91
Wang, J-Y 359
Wang, L-Y 966
Wang, R 446
Wang, Y-T 529
Wämberg, F 691
Ward III, JE 449
Warmuth-Metz, M 945
Warner, N 716
Watanabe, I 1454
Watanabe, S 1370
Watanabe, J 81
Waters, JS 481
Waters, R 1386
Watson, PH 1411
Watson, SA 567
Watters, AD 654
Webley, S 600
Weiderpass, E 324, 1417
Welslau, M 729
Werner, JA 711, 938
West, DW 54
Westphal, S 945
Weytjens, R 1437
Whitley, GStJ 673
Wiegant, J 202
Wijnen, JTh 892
Williams, RD 1445
Williams, S 905
Williamson, R 491
Wilson, P 850
Wilson, G 393
Winther, JF 524
Wistuba, II 432
Wittekind, C 689
Wittenberg, G 840
Witzigmann, H 689
Wolf, M 212
Wolfe, MM 574
Wolff, JEA 1086
Wolff, JEA 945
Wong, C-M 982
Wong, WK 1034
Wong, P 238
Workman, P 783
Wright, EP 1099
Wu, AH 54
Wu, C-H 1000
Wu, D-C 529
Wu, D-M 966
Wu, M-T 529
Wyke, AW 1128

Yaffe, M 876
Yamada, A 1006
Yamada, M 258
Yamada, Y 751
Yamagishi, H 1153
Yamamoto, N 258
Yamana, H 796
Yamazaki, K 751, 918
Yamori, T 918
Yang, B-C 359
Yang, J-Y 187
Yao, M 574
Yashima, K 441
Yasuoka, R 1153
Yates, RA 1354
Yatsuya, H 309
Ye, Z 977
Yeh, C-S 1000
Yin, X-I 218
Yip, PSF 982
Yoo, KY 314
Yoon, K-A 187
Yoon, Y-B 187
Yoshida, T 1454
Yoshimura, T 309
Yoshimura, T 37
You, S-L 966
Young, BD 537, 779
Young, MA 502
Yousef, GM 1287, 1294
Yu, C 202
Yu, J 91

Zagari, M 1341
Zannoni, GF 1145
Zappa, M 65
Zeleniuch-Jacquotte, A 49
Zhang, FW 805
Zhao, L 465
Zhou, L-F 218
Zhu, JL 524
Ziegler, J 301
Ziegler, RG 54
Zinke, J 689
Zipfel, PF 1119
Zocchi, G 497
Zoli, W 868
Zucker, JM 1197

© 2002 Cancer Research UK
